# Multiple factors influence the morphology of the bipolar electrogram: An *in silico* modeling study

**DOI:** 10.1371/journal.pcbi.1006765

**Published:** 2019-04-05

**Authors:** Minki Hwang, Jaehyuk Kim, Byounghyun Lim, Jun-Seop Song, Boyoung Joung, Eun Bo Shim, Hui-Nam Pak

**Affiliations:** 1 Division of Cardiology, Yonsei University Health System, Seoul, Republic of Korea; 2 Department of Mechanical and Biomedical Engineering, Kangwon National University, Chuncheon, Kangwon-do, Republic of Korea; University of California San Diego, UNITED STATES

## Abstract

Although bipolar electrograms (Bi-egms) are commonly used for catheter mapping and ablation of cardiac arrhythmias, the accuracy and reproducibility of Bi-egms have not been evaluated. We aimed to clarify the influence of the catheter orientation (CO), catheter contact angle (CA), local conduction velocity (CV), scar size, and catheter type on the Bi-egm morphology using an *in silico* 3-dimensional realistic model of atrial fibrillation. We constructed a 3-dimensional, realistic, *in silico* left atrial model with activation wave propagation including bipolar catheter models. Bi-egms were obtained by computing the extracellular potentials from the distal and proximal electrodes. The amplitude and width were measured on virtual Bi-egms obtained under different conditions created by changing the CO according to the wave direction, catheter-atrial wall CA, local CV, size of the non-conductive area, and catheter type. Bipolar voltages were also compared between virtual and clinically acquired Bi-egms. Bi-egm amplitudes were lower for a perpendicular than parallel CO relative to the wave direction (p<0.001), lower for a 90° than 0° CA (p<0.001), and lower for a CV of 0.13m/s than 0.48m/s (p<0.001). Larger sized non-conductive areas were associated with a decreased bipolar amplitude (p<0.001) and increased bipolar width (p<0.001). Among three commercially available catheters (Orion, Pentaray, and Thermocool), those with more narrowly spaced and smaller electrodes produced higher voltages on the virtual Bi-egms (p<0.001). Multiple factors including the CO, CA, CV, and catheter design significantly influence the Bi-egm morphology. Universal voltage cut-off values may not be appropriate for bipolar voltage-guided substrate mapping.

## Author summary

Cardiac arrhythmias are rhythm disorders of the heart leading to abnormal heart function. For the diagnosis and treatment of the arrhythmias, clinicians insert catheters into the heart and examine the electrical signal propagation in the heart. Among different type of catheters, bipolar catheters have two electrodes at the tip of the catheter with the signal being the difference between the two electrodes, which provides sharper signal than unipolar catheter. However, bipolar electrogram is dependent on many factors including catheter design and orientation, and consequently, knowledge of the determinants of the bipolar electrogram is needed for proper interpretation of the signal. In this study, we examined the effects of many factors on bipolar electrogram using computer simulation. Computer simulation is very useful in this type of study because, in clinical settings, it is not feasible to control each factor precisely. We quantitatively demonstrated the effects of catheter design and orientation, and cardiac wave propagation speed on bipolar electrogram.

## Introduction

Bipolar catheters are commonly used for electrical mapping of cardiac substrates, with an application for the diagnosis and treatment of various cardiac arrhythmias. Low-voltage areas identified based on the bipolar electrograms (Bi-egms) typically correspond to scarred tissue and its border, and are frequently targeted for intervention [1–5]. Bipolar catheters are often preferred to unipolar ones because they provide electrophysiological information regarding the area covered by two metal electrodes, producing sharp electrograms. While bipolar catheters are commonly used in clinical settings, the Bi-egm morphology is known to depend on many factors including the catheter orientation relative to the direction of the activation wave [6–9], catheter contact angle [7], conduction velocity (CV) [8], and catheter size and shape [8,10–12]. There have been attempts to understand the role of each such factor and develop a strategy to interpret the Bi-egms accordingly. It has been reported that the bipolar voltage increases as the catheter orientation relative to the activation wave direction changes from perpendicular to longitudinal, and decreases as the CV decreases [8]. Blauer et al. [7] used *in silico* modeling and simulation to examine the effects of the catheter orientation and contact angle on the accuracy of voltage mapping, and found that a higher contact angle with respect to the tissue may improve the voltage mapping accuracy. In the present study, we focused on the Bi-egm morphology clarifying the effects of each aforementioned factor using *in silico* modeling and simulation. In experimental or clinical studies, it is not feasible to reproducibly examine the effects of any single factor on the Bi-egm morphology while keeping other factors unchanged. On the other hand, *in silico* simulation enables a systematic quantification of the effects of each factor while controlling for all other factors. Importantly, we simulated three commercially available bipolar catheters and compared their Bi-egm amplitudes in various catheter orientations and contact angles. Our present findings confirmed the clinical observations regarding the effect of the various factors on the Bi-egm morphology and provided insight into the mechanism of the Bi-egm generation, which may be useful for a proper interpretation of the Bi-egm.

## Results

### Effect of the catheter orientation, contact angle, and CV on the Bi-egm morphology

**Fig 1:**
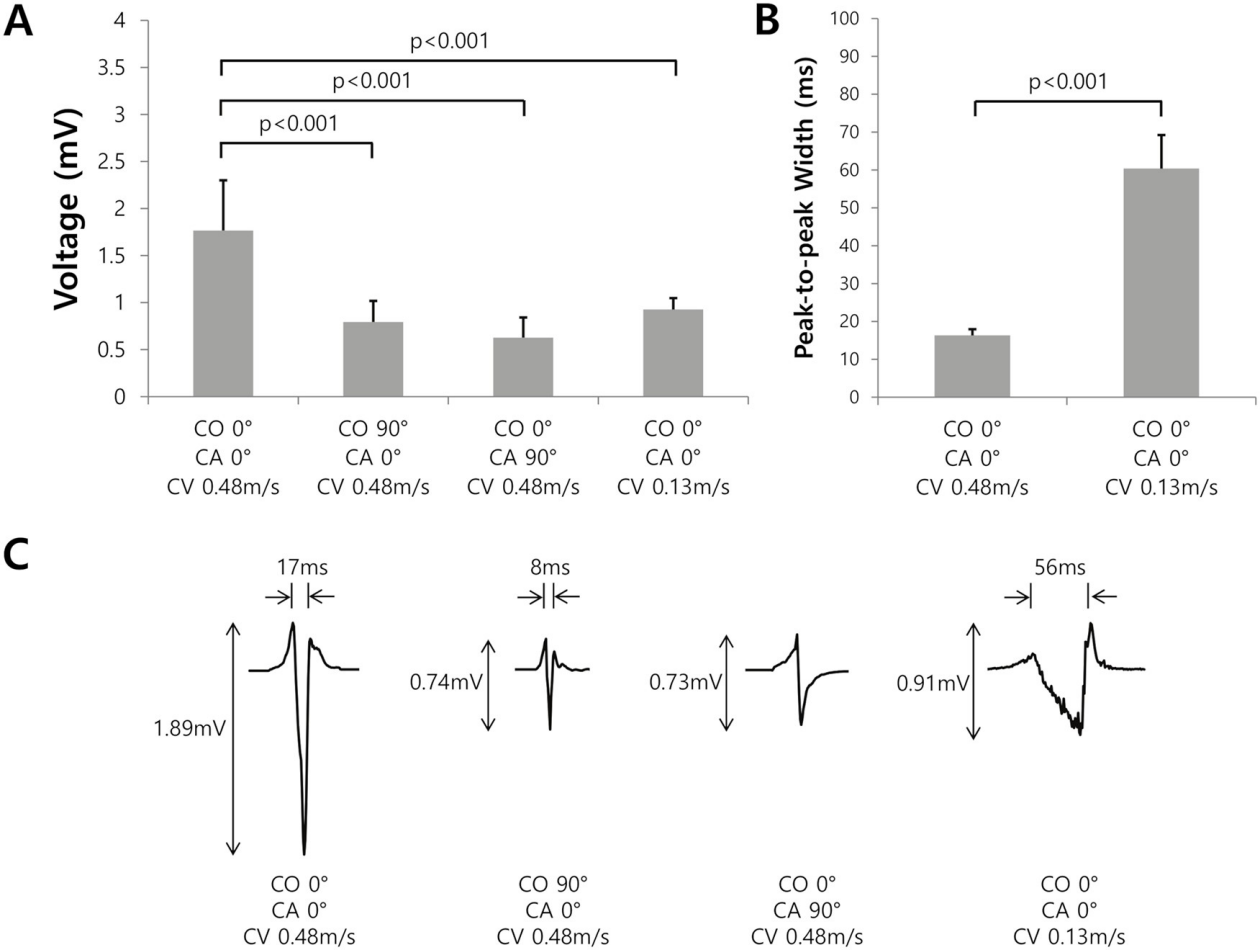
Bipolar voltage and peak-to-peak width for different catheter orientations (COs), contact angles (CAs), and conduction velocities (CVs). A. Bipolar voltage change with an increasing CO, increasing CA, and decreasing CV. B. Peak-to-peak widths for the different CVs. C. Representative examples of the bipolar electrogram for the different COs, CAs, and CVs.

As the catheter orientation of the virtual Thermocool catheter changed from 0° to 90°, the bipolar amplitude decreased from 1.77±0.53 to 0.79±0.22 mV (p<0.001; Fig 1A), while the peak-to-peak width decreased from 16±1 to 9±5 ms (p<0.001). Furthermore, as the catheter contact angle of the virtual Thermocool catheter changed from 0° to 90°, the amplitude decreased from 1.77±0.53 to 0.63±0.21 mV (p<0.001; Fig 1A). These Bi-egms exhibited a single positive peak and single negative peak because the magnitude of the signal from the proximal electrode was relatively small due to the relatively long distance between the proximal electrode and atrial surface. As the CV decreased from 0.48 to 0.13 m/s, the bipolar amplitude decreased from 1.77±0.53 to 0.93±0.12 mV (p<0.001; Fig 1A), while the peak-to-peak width increased from 16±1 to 60±9 ms (p<0.001; Fig 1B). Representative Bi-egms for different catheter orientations, contact angles, and CVs are provided in Fig 1C.

### Effect of AF and the APD_90_ on the Bi-egm morphology

**Fig 2:**
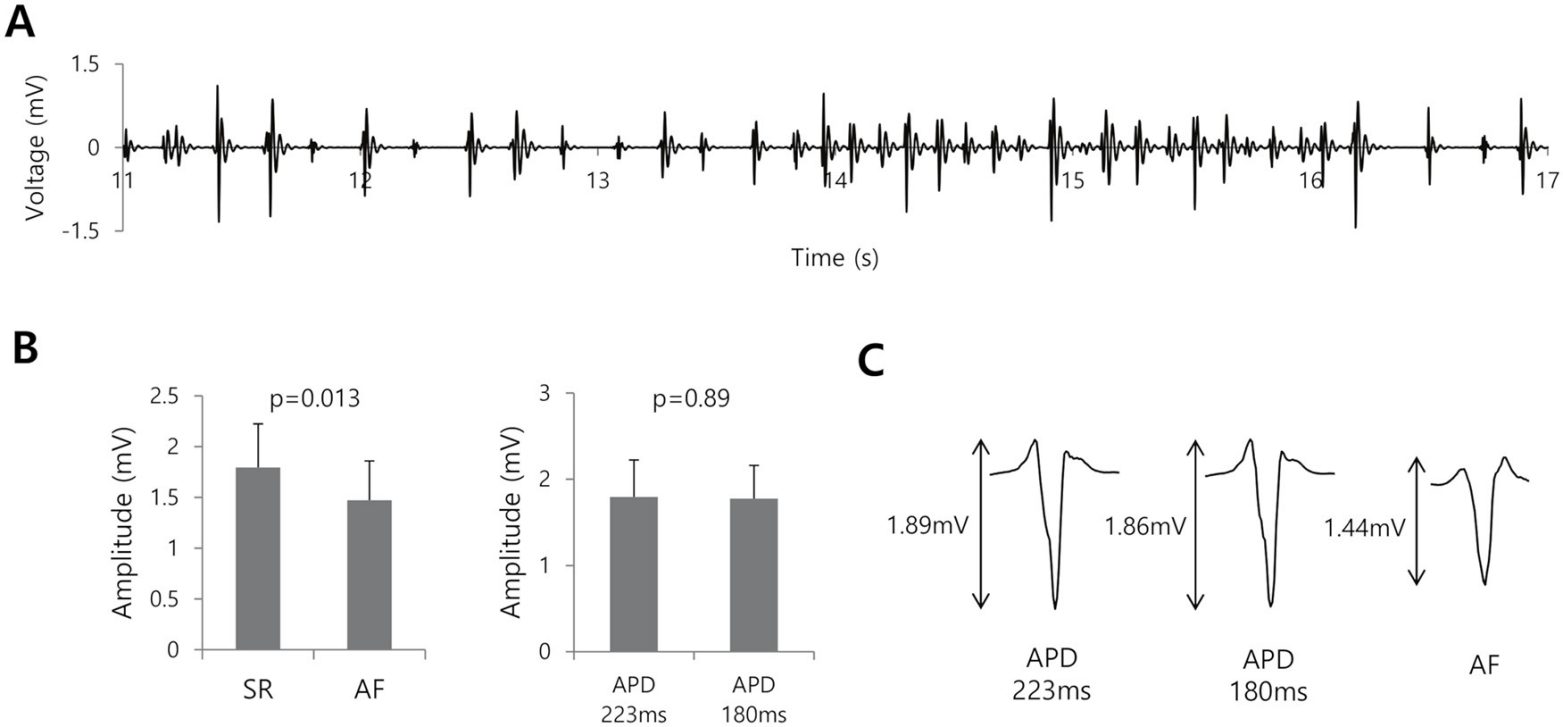
Bipolar electrogram signal amplitude during atrial fibrillation (AF) and with different action potential durations at 90% repolarization (APD_90_). A. Representative bipolar electrogram under AF conditions with a bandpass filter setting of 30–150 Hz. B. Bipolar amplitudes and widths under sinus rhythm (SR) and AF conditions. C. Bipolar amplitudes and widths for the different APD_90_ at a pacing cycle length of 600ms. D. Representative bipolar electrograms for the different APD_90_s and under AF conditions.

A representative Bi-egm strip obtained for the virtual Thermocool catheter in the 3D atrial model with induced AF is provided in Fig 2A. Compared to the sinus rhythm conditions, the AF conditions produced a lower bipolar amplitude (1.77±0.53 vs. 1.47±0.39 mV, p = 0.013; Fig 2B). That seemed to be due to the random direction of the activation wave with respect to the catheter orientation. On the other hand, the bipolar amplitude did not change significantly upon reducing the APD_90_ from 223 to 180 ms (Fig 2B). Representative Bi-egms for the different APD_90_ and under AF conditions are provided in Fig 2C.

### Effect of the scar size and catheter type

**Fig 3:**
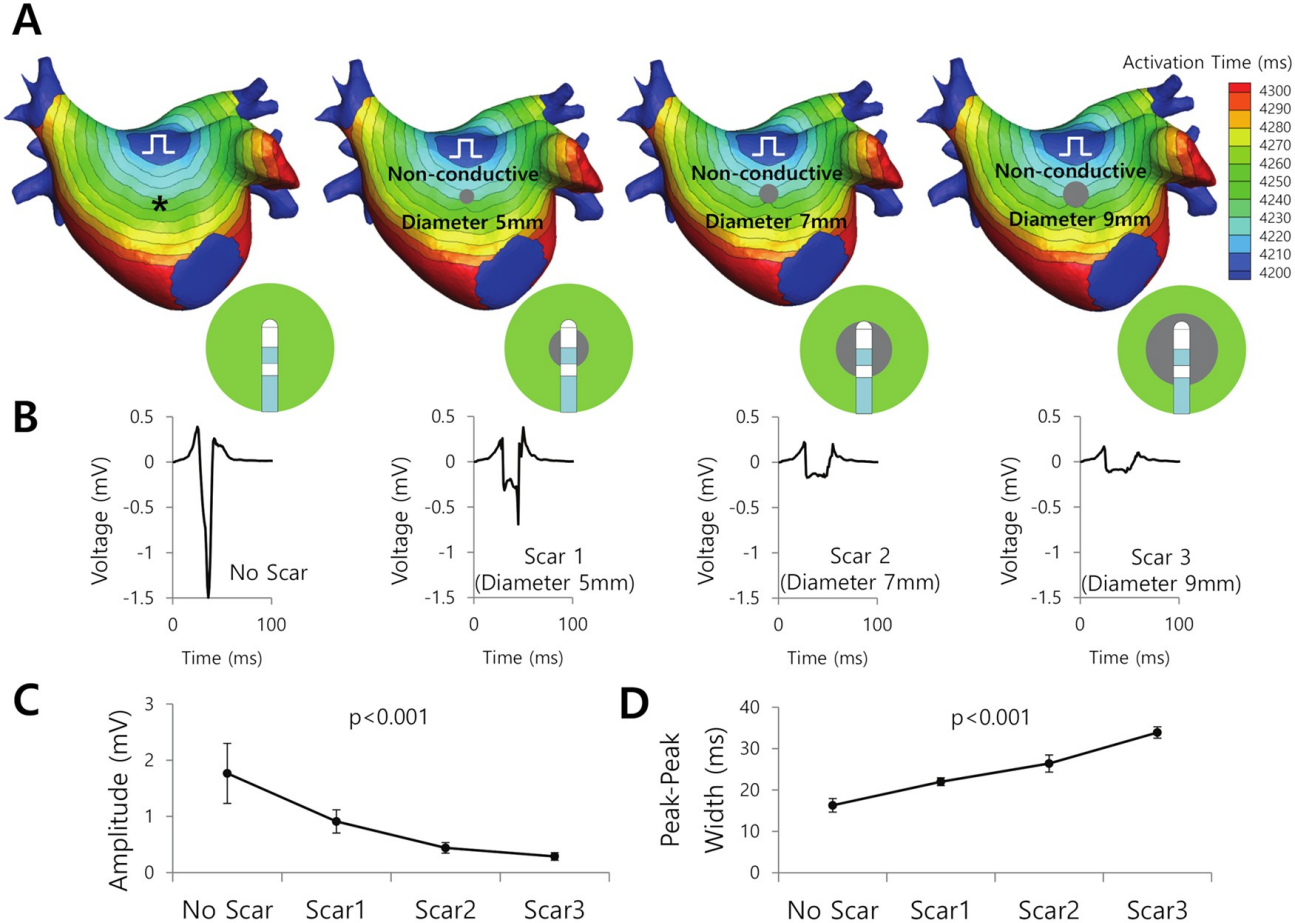
Bipolar amplitudes and widths for the different scar sizes. Scar tissue was simulated as a non-conductive area. A. The location of the scar (grey color), position of the virtual catheter with respect to the scar, and activation time map are shown. The symbol (⎍) indicates pacing site. B. Representative bipolar electrograms of scars of different sizes. Scars 1, 2, and 3 were simulated as non-conductive circular areas with a diameter of 5, 7, and 9mm, respectively. C. The bipolar amplitude according to the scar size. D. Peak-to-peak width according to the scar size.

Virtual scars were modeled as circular non-conductive areas with a diameter of 5, 7, or 9 mm on the anterior side of the left atrial model (Fig 3A). The activation time map and positions of the virtual Thermocool catheter relative to the virtual scars are also shown in Fig 3A. Representative Bi-egms for each size of scar are provided in Fig 3B. The bipolar amplitude decreased (p<0.001; Fig 3C) while the peak-to-peak width increased (p<0.001; Fig 3D) as the scar diameter increased from 0 to 9mm.

**Fig 4:**
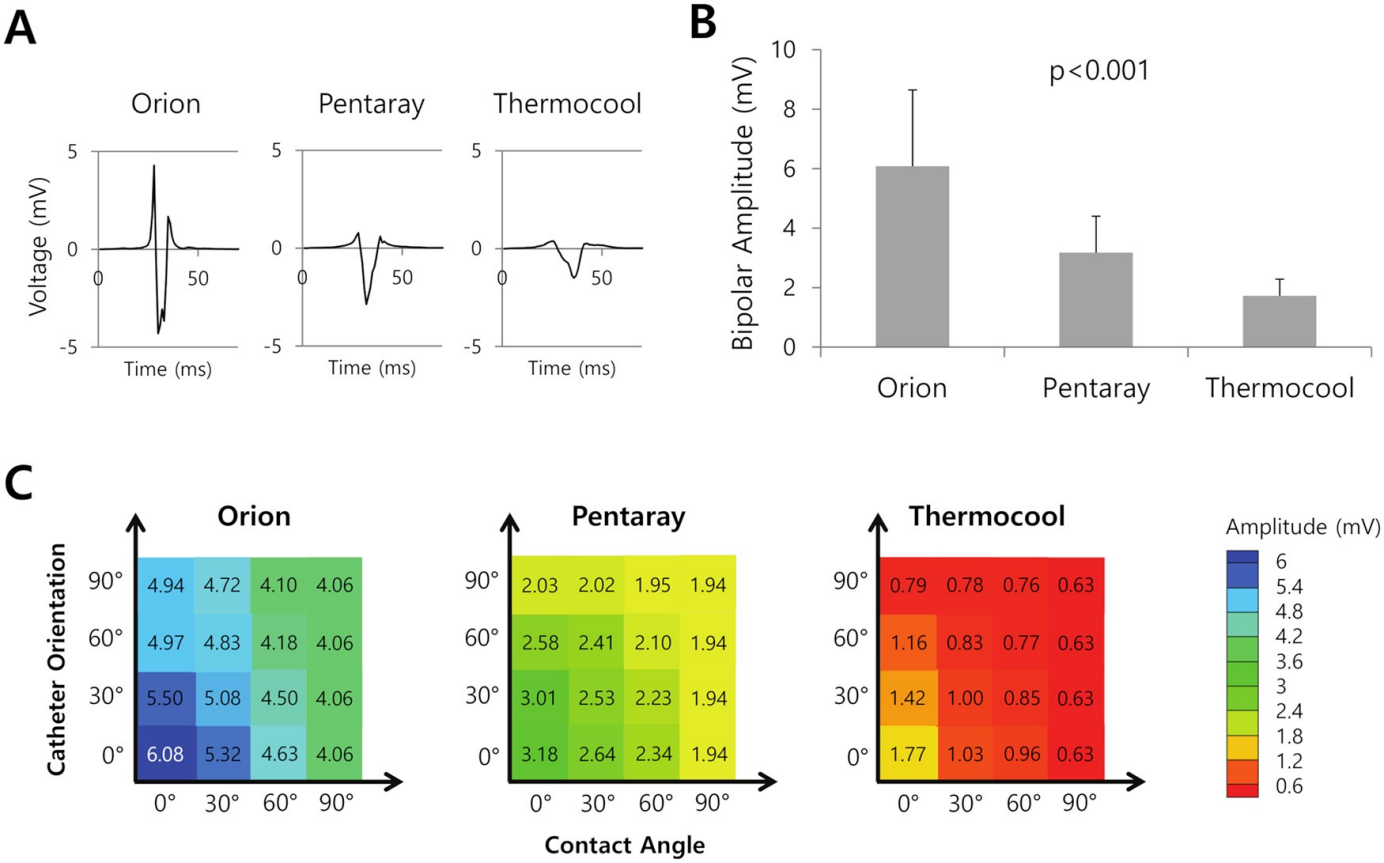
Bipolar amplitude according to the type of catheter. A. Representative bipolar electrograms obtained at the location marked with an asterisk in Fig 3A. The location was randomly selected in the anterior region of the left atrium. B. Bipolar amplitudes for the Orion, Pentaray, and Thermocool catheters. C. Color maps of the bipolar amplitude for the Orion, Pentaray, and Thermocool catheters in catheter orientations of 0°, 30°, 60°, and 90° and catheter contact angles of 0°, 30°, 60°, and 90°.

Three types of catheters corresponding to commercially available catheters (Thermocool, Pentaray, and Orion) were tested. The representative Bi-egms for each catheter type are provided in Fig 4A. The bipolar amplitude was highest for the Orion catheter, followed by the Pentary and Thermcool catheters (p<0.001; Fig 4B). The contact with the atrial surface covered the entire electrode area of the Orion catheter because the electrodes of this catheter are flat; on the other hand, only portions of the electrodes of the Thermocool and Pentaray catheters contacted the atrial surface because the electrodes of those catheters are cylindrical. The shape and size of the electrode are the most likely factors explaining the high electrode signal provided by the Orion catheter compared to that provided by the other catheters. Upon examining the Bi-egms for the three catheter types at catheter orientations and contact angles of 0°, 30°, 60°, and 90°, we found that the bipolar amplitude decreased as the catheter orientation and/or contact angle increased (Fig 4C).

### Comparison of virtual and clinical voltage maps

**Fig 5:**
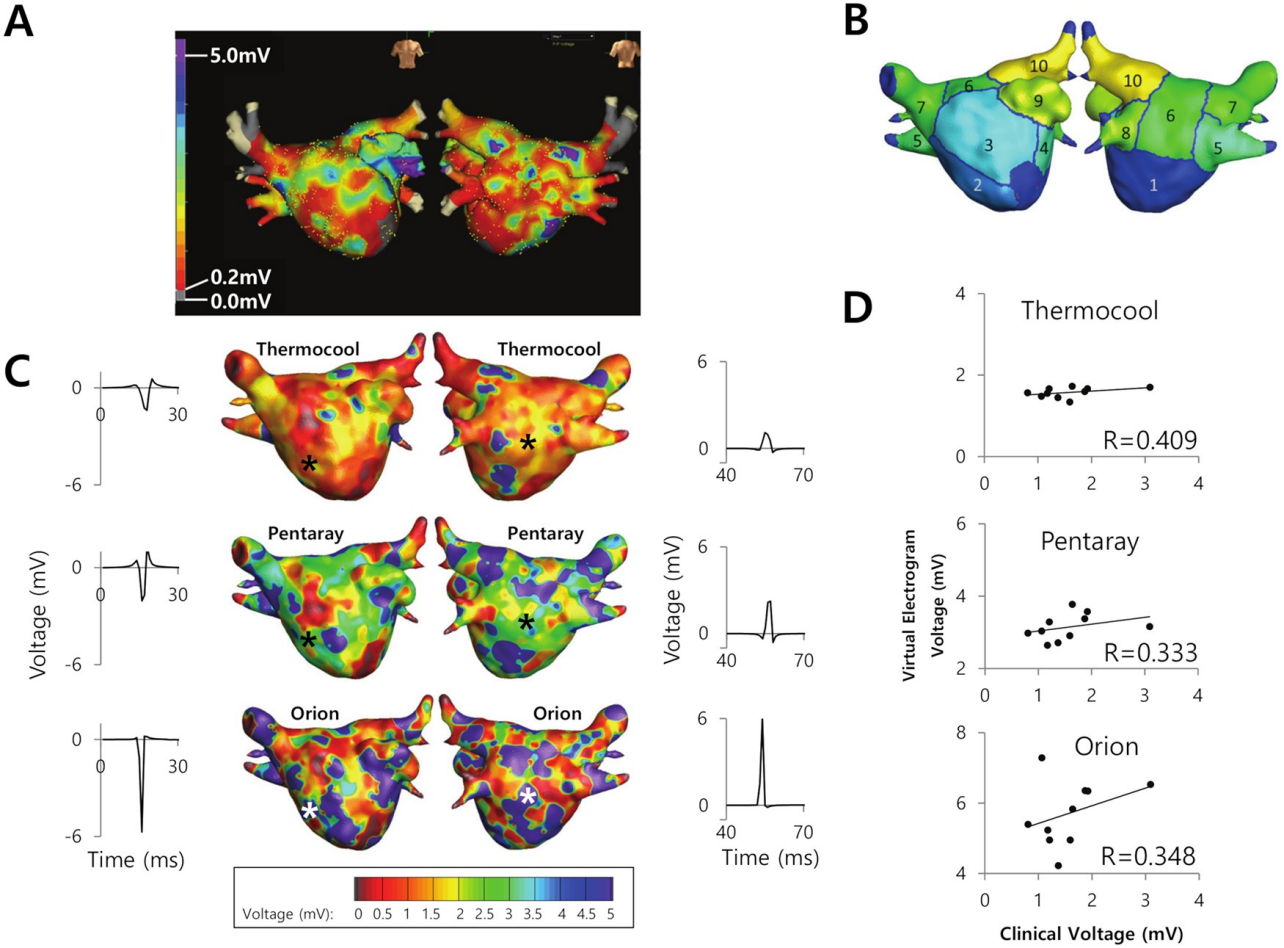
Comparison of clinically acquired and virtual voltage maps. A. Voltage map acquired in a clinical setting using a ring catheter with 1.0-mm electrodes spaced at 3.0 mm (AFocus, Abbott, Lake Bluff, IL, USA). B. Segmentation of the left atrium model into 10 segments for the calculation of the bipolar voltage. C. Virtual voltage maps for the Thermocool, Pentaray, and Orion catheters (from top to bottom). Electrograms were obtained at the locations marked with an asterisk. D. Correlation between the clinically acquired and virtual voltages acquired with the Thermocool, Pentaray, and Orion catheters (from top to bottom).

A clinically acquired voltage map is illustrated in Fig 5A. To facilitate a quantitative comparison between the clinically acquired and virtual maps, the left atrium was divided into 10 segments (Fig 5B). The virtual voltage maps obtained for each type of bipolar catheter (Fig 5C) were compared against the clinically acquired map. The average voltage was higher for the virtual Orion and Pentaray catheters than the Thermocool catheter (5.71±0.92 and 3.15±0.36 mV, respectively, vs. 1.57±0.12 mV; p<0.001 for each comparison). The average voltage in each of the 10 segments of the left atrium (Fig 5B) showed a moderate correlation between the virtual and clinically acquired data (r = 0.409 for Thermocool, r = 0.333 for Pentaray, and r = 0.348 for Orion; Fig 5D).

### Sensitivity analysis

**Fig 6:**
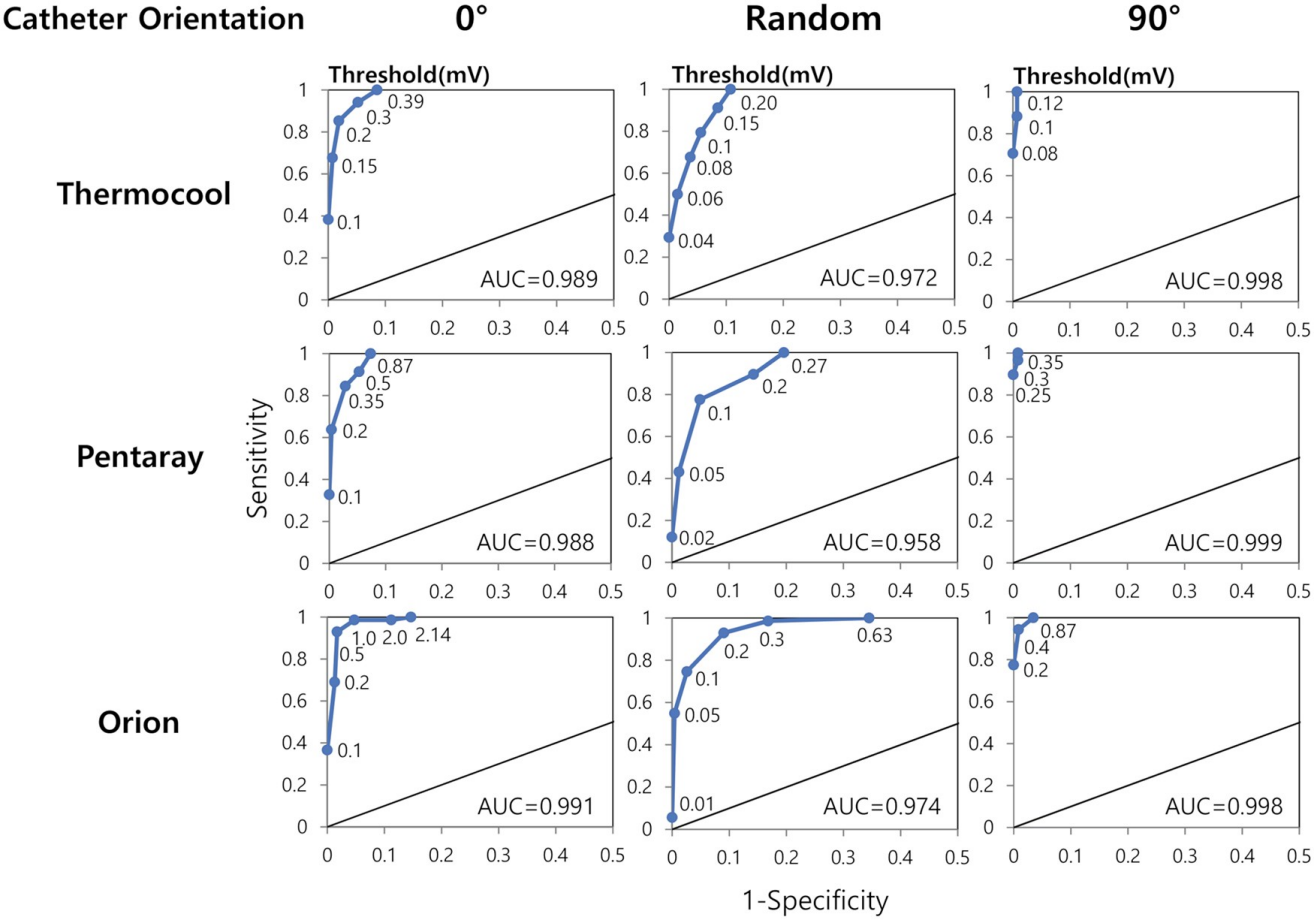
Sensitivity analysis. Sensitivity analysis was performed on the lower cut-off voltages to differentiate scar area for three types of catheters (Thermocool, Pentaray, and Orion) with three orientations of catheter with respect to the wave direction: 0°, 90°, and random angles.

Fig 6 shows the sensitivity analysis on the lower cut-off voltages to differentiate scar area for the three types of catheters (Thermocool, Pentaray, and Orion). When catheter orientations were randomly selected, all the catheters in the scar area showed voltages less than 0.20, 0.27, and 0.63 mV for Thermocool, Pentaray, and Orion catheters, respectively. When catheter orientations were parallel to the wave direction, all the catheters in the scar area showed voltages less than 0.39, 0.87, and 2.14 mV for Thermocool, Pentaray, and Orion catheters, respectively. When catheter orientations were perpendicular to the wave direction, all the catheters in the scar area showed voltages less than 0.12, 0.35, and 0.87 mV for Thermocool, Pentaray, and Orion catheters, respectively (Fig 6).

## Discussion

### Main findings

In this study, we simulated the Bi-egm generation applying virtual bipolar catheters mimicking three commercially available catheters to examine the activation wave propagation in a realistic model of the left atrium. The factors associated with a reduced bipolar amplitude included an increased catheter orientation, increased contact angle, and decreased CV. Furthermore, the bipolar amplitude decreased under AF conditions. On the other hand, the change in the APD_90_ was not associated with a significant change in the bipolar amplitude. Increasing scar size was associated with a decreased bipolar amplitude and increased peak-to-peak width. Among the three types of commercially available bipolar catheters (Thermocool, Pentaray, and Orion), the Orion catheter produced the greatest bipolar amplitude followed by the Pentaray and Thermocool catheters. There was a moderate correlation between the clinically acquired and virtual bipolar voltages in the left atrium.

### Factors affecting the Bi-egm morphology

It has long been recognized that the Bi-egm morphology is affected by multiple factors including the catheter orientation, contact angle, and catheter geometry [6–12]. With the bipolar catheter, the two electrodes lie at a certain distance from each other, which inevitably causes the electrodes to sense the activation wave at different times depending on the catheter orientation [13] and on the inter-electrode spacing, with a greater spacing leading to a greater difference between the times at which the activation wave arrives at each electrode. Moreover, at contact angles greater than 0°, the signal intensity is stronger at the distal electrode than at the proximal electrode, which also contributes to the variation in the Bi-egm morphology. On the other hand, an increased CV is expected to decrease the difference between the times at which the wave arrives at the electrodes, regardless of the catheter orientation and contact angle. The catheter size and shape are also known to affect the Bi-egm morphology. Among the three commercially available catheter types tested in this study, the signal amplitude was smallest for the Thermocool catheter, possibly due to the specific size and shape of the electrodes used on that catheter. In the case of cylindrical electrodes, increasing the electrode size results in an increased absolute but not relative contact area with the atrial surface because the opposite side of the electrode does not contact the atrial surface and is moreover located farther from the atrial surface, which results in lower signal amplitude upon averaging over the entire electrode.

### Identification of the scar area based on the Bi-egms

The Bi-egm is commonly used for electrical mapping of the heart to identify scar areas that will be targeted for catheter ablation of arrhythmias. In this study, we confirmed that the signal amplitude from the mapping catheter is decreased in scar areas, and noted that the decrease is proportional to the scar size. Additionally, all geometrical factors affecting the Bi-egm morphology in the absence of a scar appear to have a similar effect in the presence of a scar. Thus, in clinical practice, low-voltage areas may indicate the presence of scar or may reflect an inappropriate orientation of the catheter. The findings of the *in silico* study by Blauer et al. [7], which investigated the effect of the contact angle on the ability to identify scar areas via catheter mapping are informative in this context. As our present results confirmed, the catheter size is especially important for the ability to detect scar areas. Specifically, smaller electrodes tend to produce a higher voltage, whereas bigger catheters are more likely to cover the border of the scar and thus reflect attenuated values. Anter et al. [12] reported that the low-voltage area was larger and the mean voltage within the low-voltage area was lower when measured using a linear catheter with a 3.5-mm distal electrode than when measured using a 1-mm multielectrode-mapping catheter in patients with scar-related atrial arrhythmias. Taken together, the present findings and previous observations confirm the fact that different catheter types produce different voltage values, suggesting that it may be necessary to develop catheter-specific voltage criteria for a scar definition of electrical mapping.

### Clinical implications

Traditionally, electrophysiology studies for arrhythmia mapping in the clinical setting have used activation, pace, and entrainment mapping methods [14–16]. However, if the activation sequence of the reentrant tachycardia is constantly changing or degenerating to chaotic fibrillation, it is difficult to map the driver of the arrhythmia using conventional approaches. In such cases, Bi-egm voltage-based substrate mapping, which can be acquired during sinus rhythm or with regular pacing, helps identify low-voltage areas corresponding to scar or its border zone and is useful for locating the slow conduction zone [4]. However, a substrate map based on the local bipolar voltage is influenced by multiple factors [6–12], as was also shown in our present study. In the clinical electrophysiology laboratory, it is not feasible to generate homogeneous substrate maps with the same contact force, catheter orientation, and tissue conditions. The local CV should differ depending on the substrate remodeling and fibrosis. Moreover, under AF conditions, the catheter orientation relative to the wave direction cannot be controlled when conducting point-by-point mapping using single or multi-electrode catheters. Nevertheless, interventional electrophysiologists are using empirical voltage cut-off values to define low-voltage areas indicative of scar or scar border zones [4], and it is clear that bipolar voltage-based substrate mapping provides very important information during complex arrhythmia mapping. However, 3D substrate mapping using the current technology employs spatiotemporal assumptions, and the operator should consider the various factors affecting the B-egm morphology. In particular, radiofrequency ablation guided by a 3D color substrate map alone may misidentify the target. Thus, simultaneous and careful monitoring of the electrograms provided by the ablation catheter tip are expected to facilitate a successful ablation.

### Limitations

This study had some limitations. The 3D atrial model used for simulating the propagation of the activation wave was homogeneous in its thickness and did not consider transmural variations in the activation wave propagation. Another limitation was that this study did not test the mapping accuracy of the scar area for different types of catheters, which represented our focus for further research.

### Conclusion

Multiple factors including the catheter orientation, contact angle, CV, and catheter design have a significant influence on the Bi-egm morphology. It may not be appropriate to use a universal cut-off value for the bipolar voltage during voltage-guided substrate mapping.

## Methods

### Electrical wave propagation model

Computed tomography images of the human left atrium were segmented to generate a 3-dimensional (3D) model of the left atrium using the NavX system (Abbott, Lake Bluff, IL, USA). A triangular mesh was generated on the atrial model for the calculation of the electrical potentials. The electrical wave propagation in the atrium was simulated by numerically solving the following reaction-diffusion equation [17]:

$$\frac{\partial V_{m}}{\partial t}=\frac{1}{\beta C_{m}}\left\{\nabla \cdot D\nabla V_{m}-\beta \left(I_{ion}+I_{s}\right)\right\}$$ (1)

where *Vm* is the membrane potential, β is the membrane surface-to-volume ratio, C_m_ is the membrane capacitance per unit area, D is the conductivity tensor, and I_ion_ and I_s_ are, respectively, the ionic and stimulation currents. A mathematical model of the human atrial I_ion_, as developed by Courtemanche et al. [18], was adopted to determine the ionic currents at each computational node. For the I_s_, a current of -2900pA was applied for 1.5ms at each pacing time at a site corresponding to the location of Bachmann’s bundle.

### Virtual catheter

**Fig 7:**
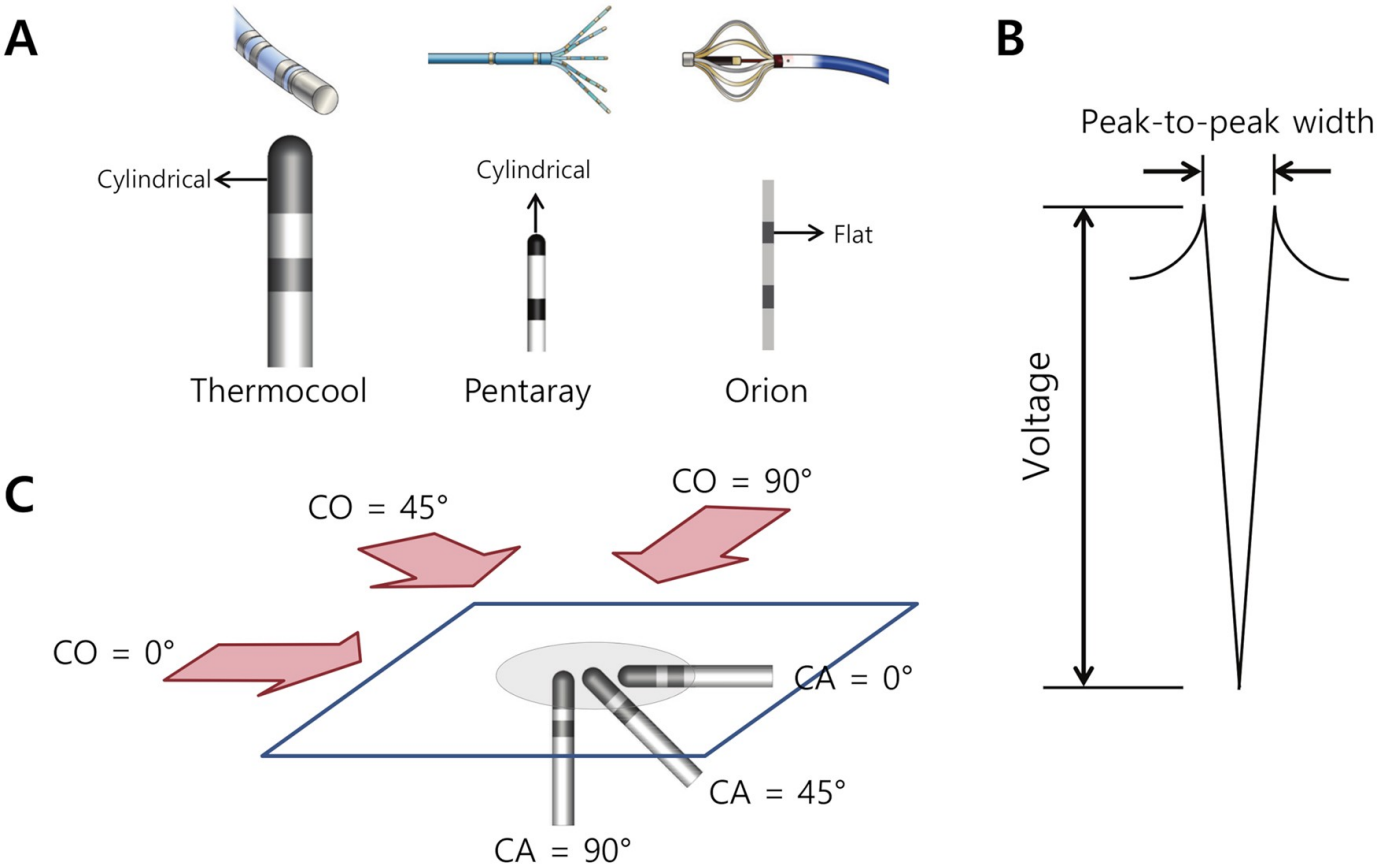
Bipolar catheter models and definitions of the catheter orientation (CO) and catheter contact angle (CA). A. Models of three commercially available bipolar catheters. B. Schematic of the bipolar electrogram. The bipolar voltage and peak-to-peak width are indicated. C. The CA was defined as the angle between the catheter axis and the atrial surface. The CO was defined as the angle between the direction of the activation wave and the catheter axis at a CA of 0°.

Models of three commercially available catheters were constructed by creating virtual objects with the corresponding shape and dimensions (Fig 7A): (i) a virtual Thermocool catheter with cylindrical electrodes having a 3.5-mm tip and 2-mm inter-electrode spacing (Thermocool; Biosense Webster, Diamond Bar, CA, USA); (ii) a virtual Pentaray catheter with cylindrical electrodes having a 1-mm tip and 2-mm inter-electrode spacing (Pentaray; Biosense Webster, Diamond Bar, CA, USA); and (iii) a virtual Orion catheter with flat electrodes having a 0.4-mm^2^ electrode area and 2.5-mm inter-electrode spacing (Intellamap Orion; Boston Scientific, Marlborough, MA, USA). The voltage measured by each of the two electrodes of the bipolar catheter was obtained by computing the extracellular potential for the respective electrode [19]. To examine the effects of the size and shape of the electrode on the Bi-egm morphology, the part of the model corresponding to each electrode was divided into many smaller regions (~0.07mm^2^). The extracellular potentials were computed for each of these smaller areas and then averaged to obtain the voltage of the whole electrode. The Bi-egm voltage was obtained as the difference between the voltages noted for the proximal and distal electrodes, with a sampling frequency of 1kHz.

### Virtual measurement of the Bi-egm

To quantify the effects of the catheter orientation and contact angle on the Bi-egm morphology, the virtual catheter was placed on the surface of the 3D model of the atrium, and an electrical stimulus was applied at the location of Bachmann’s bundle using a pacing cycle length (PCL) of 600ms. The bipolar voltage and peak-to-peak width were measured as shown in Fig 7B for various catheter orientations and contact angles (as defined in Fig 7C). For a catheter orientation and contact angle of 0°, CVs of 0.48 and 0.13m/s were tested. The effect of the action potential duration at 90% repolarization (APD_90_) was also tested at a PCL of 600ms; two APD_90_ values were tested (223 ms and 180 ms). The APD_90_ was changed by adjusting the rapid and slow delayed rectifier K^+^ currents. To examine the Bi-egm morphology in atrial fibrillation (AF), virtual AF was induced as described previously [20]. The bipolar voltage was measured in AF for 6 s. To examine the effect of the scar size on the Bi-egm morphology, a virtual scar was modeled as a circular non-conductive area, and three diameters were tested (5, 7, and 9mm). The virtual bipolar catheter was placed at the location of the virtual scar, and the Bi-egm morphology was examined at a PCL of 600ms.

### Fibrosis modeling

The location of fibrotic areas was determined based on a clinically acquired bipolar voltage map. First, the bipolar voltage data from the clinically acquired map were interpolated to the computational nodes on the 3D *in silico* atrial model. To determine the fibrosis status (yes/no) for each node, we used the following nonlinear relation between the bipolar voltage and probability of fibrosis.

$$\text{Y}=-40.0{\text{X}}^{3}+155{\text{X}}^{2}-206\text{X}+99.8$$ (2)

where Y is the probability that there is fibrosis at a given node, whereas X is the bipolar voltage at that node. Eq (2) was developed by comparing the predicted percentage of fibrosis across the 3D atrial model with the pre- and post-ablation fibrosis data reported in the literature [21] for patients with paroxysmal or persistent AF. For each node, the probability of fibrosis calculated based on clinically acquired bipolar voltage data (Eq (2)) was compared against a random number between 0 and 1. If the random number was below the calculated probability of fibrosis, the node was considered to have a positive fibrosis status. The ionic currents and diffusion coefficients in the fibrosis area were adjusted as described elsewhere [22].

### Virtual voltage map generation

To generate virtual bipolar voltage maps, the activation wave propagation in the 3D atrial model was simulated at a PCL of 600ms. The virtual bipolar catheter was placed at 810 randomly selected nodes with a random orientation of the catheter. The virtual bipolar voltage was calculated at the 810 nodes, and those voltages were interpolated to all computational nodes to generate the virtual bipolar voltage map. Such a voltage map was obtained for the three types of virtual catheters (Thermocool, Pentaray, and Orion). The 3D atrial model was divided into 10 sections, and the average voltage in each section was compared between the clinically acquired and virtual maps.

### Sensitivity analysis

A sensitivity analysis was performed on the lower cut-off voltages to differentiate scar area for the three types of catheters (Thermocool, Pentaray, and Orion). A circular non-conductive zone (Diameter = 18mm) was created in the anterior region of a 3-dimensional model of the left atrium. Virtual catheter was placed at random locations in and around the non-conductive zone (N = 303). The numbers of locations at which both of the distal and proximal electrodes were fully included in the non-conductive zone were 34, 58, and 71 for Thermocool, Pentaray, and Orion catheters, respectively. An electrical stimulation was applied at a site corresponding to the location of Bachmann’s bundle with PCL of 600ms. Bipolar voltage was obtained at the eighth stimulation. Three catheter orientations with respect to the wave direction were tested: 0°, 90°, and random angles. Sensitivity analysis was performed with various threshold voltages until the voltages of all the locations in the scar area were less than the threshold voltage.
